# A risk scoring system to predict the individual incidence of early-onset colorectal cancer

**DOI:** 10.1186/s12885-022-09238-4

**Published:** 2022-01-29

**Authors:** Jialin Gu, Yan Li, Jialin Yu, Miao Hu, Yi Ji, Lingchang Li, Canhong Hu, Guoli Wei, Jiege Huo

**Affiliations:** 1 Department of Oncology, Affiliated Hospital of Integrated Traditional Chinese and Western Medicine, Nanjing University of Chinese Medicine, 100 Cross Street, Maigaoqiao, Nanjing, Jiangsu 210028 P.R. China; 2 Nanjing University of Chinese Medicine, Nanjing, 210046 Jiangsu China; 3 Department of Oncology, Jiangsu Province Academy of Traditional Chinese Medicine, Nanjing, 210028 Jiangsu China; 4 Nanjing Lishui District Hospital of Traditional Chinese Medicine, Nanjing, 211200 Jiangsu China; 5 Yangzhou University Medical College, Yangzhou, 225000 Jiangsu China

**Keywords:** Early-onset colorectal cancer, Risk factors, Meta-analysis, Rothman–Keller model, Risk assessment

## Abstract

**Background:**

The incidence of early-onset colorectal cancer (EOCRC) is increasing at an alarming rate and further studies are needed to identify risk factors and to develop prevention strategies.

**Methods:**

Risk factors significantly associated with EOCRC were identified using meta-analysis. An individual risk appraisal model was constructed using the Rothman–Keller model. Next, a group of random data sets was generated using the binomial distribution function method, to determine nodes of risk assessment levels and to identify low, medium, and high risk populations.

**Results:**

A total of 32,843 EOCRC patients were identified in this study, and nine significant risk factors were identified using meta-analysis, including male sex, Caucasian ethnicity, sedentary lifestyle, inflammatory bowel disease, and high intake of red meat and processed meat. After simulating the risk assessment data of 10,000 subjects, scores of 0 to 0.0018, 0.0018 to 0.0036, and 0.0036 or more were respectively considered as low-, moderate-, and high-risk populations for the EOCRC population based on risk trends from the Rothman–Keller model.

**Conclusion:**

This model can be used for screening of young adults to predict high risk of EOCRC and will contribute to the primary prevention strategies and the reduction of risk of developing EOCRC.

**Supplementary Information:**

The online version contains supplementary material available at 10.1186/s12885-022-09238-4.

## Background

Although the incidence of colorectal cancer (CRC) has declined with the support of medical technology and prevention policies, a completely opposite trend has been observed in young adults under the age of 50 years [1, 2]. Early-onset colorectal cancer (EOCRC) is defined as colorectal cancer diagnosed before the age of 50 years, and has shown a progressively increasing incidence worldwide. Studies have reported that approximately 11% of CRC cases registered in the National Cancer Database were diagnosed in adults aged 18 to 49 years [3]. Similarly, recent data from Europe indicate that the incidence of CRC increased by 7.9, 4.9, and 1.6% per year among subjects aged 20–29, 30–39, and 40–49 years from 2004 to 2016, respectively [4]. Most cases of EOCRC are diagnosed after the onset of symptoms, which include bloody stool and abdominalgia, increasing the danger of delayed diagnosis and poor prognosis [5, 6].

The causes of the rising incidence of EOCRC have not been fully elucidated. The majority of EOCRC cases are disseminated and may be associated with changes in environmental, behavioral, and dietary patterns. Several studies have reported an increased risk of EOCRC from alcohol consumption, sedentary lifestyle, and high intake of red and processed meats [7–10]. In addition, lower levels of schooling may also increase the prevalence of EOCRC [7, 11]. Primary prevention is a key strategy to reduce the burden of this disease. The American Cancer Society has lowered the age of screening for people at risk of colorectal cancer from 50 to 45 years of age [12]. Studies demonstrate that increasing participation in population-based risk screening not only reduces mortality but also reduces health care costs [13]. Therefore, it is important to identify risk factors for EOCRC. Previous meta-analyses have identified risk factors such as family history of CRC, male sex, and obesity. However, there are still other factors that have revealed non-significant associations due to small sample sizes with insufficient statistical power [14, 15]. Due to the needs of large-scale population screening, it is essential to build an individualized risk prediction and evaluation model, which can help evaluate and identify high-risk populations for EOCRC. Previous studies have found that individualized risk-based screening is more likely to be accepted [16].

Accordingly, we established the EOCRC risk appraisal and prediction system using the Rothman–Keller model, aiming at early and effective identification of high-risk populations of EOCRC. Our scoring system also provides easy risk prediction formulas for individuals to achieve potential risk reduction.

## Methods

### Search strategy and study selection

Based on a previously published meta-analysis [14], we conducted a comprehensive search in PubMed and the Web of Science (WOS) to discover new original studies using the following terms: “colorectal cancer,” “colorectal neoplasms,” “colon tumor,” “rectum tumor,” “colon cancer,” “rectum cancer,” “early onset,” “young onset,” “young adult”, “age of 50”, “risk”. Multiple combinations of the above search terms were used. Studies that met the following criteria were considered: i) Diagnosis consistent with EOCRC, ii) cohort studies or case-control studies, and iii) control group age-matched with non-EOCRC patients of the case group. Only studies published in English were considered. Literature management and review was performed using Endnote × 9 (V9.3.3, Clarivate Analytics) [17]. References that met the inclusion criteria were manually screened to avoid omissions. Reviews, case reports, experimental studies, duplicate publications, and studies that did not meet the diagnosis of EOCRC were excluded. The titles, abstracts, and subsequent full text of the retrieved publications were screened by two independent reviewers. A third reviewer decided on any disagreements.

### Data extraction

Baseline data were collected from all patients, including sex, race, past medical history (diabetes, inflammatory bowel disease (IBD), hyperlipidemia, hypertension), dietary factors (processed meat, red meat), lifestyle habits (sedentary, obesity, alcohol intake, smoking, dessert), and medication history (aspirin and NSAIDs). Screening of risk factors for EOCRC was derived from meta-analysis and the factors that significantly correlated with EOCRC (*P* < 0.05) were included in subsequent model construction.

### Construction of the risk appraisal model

The Rothman–Keller model was used to construct an individualized risk appraisal model for EOCRC [18]. It was first applied in 1972 to assess the effects of alcohol and tobacco on the risk of oral and laryngeal cancers. It considers both independent and interactive effects of influencing factors and has been applied in the risk assessment and prevention of a multitude of chronic diseases [19]. The relative ratio (RR) can be replaced by the odds ratios (OR) when the outcome occurs in less than 10% [20, 21]. The computational procedure of Rothman–Keller model is as follows:

(I) Population attributable risk percentage (*PAR*%)

$$PAR\%=\frac{P_i\left({RR}_i-1\right)}{P_i\left({RR}_i-1\right)+1}\times 100\%$$

(II) Baseline incidence ratio (*ρ*)

$$\rho =\frac{1}{\sum_{i=1}^n{RR}_i\times {P}_i}=1- PAR\%$$

*P*_*i*_: the proportion of individuals exposed to a risk factor in the overall population; *RR*_*i*_: the relative risk of exposure to a risk factor.

(III) Risk score (*S*) and combined risk score (*θ*)

$$S=\rho \times {RR}_i$$

$$\theta =\left({M}_1-1\right)+\left({M}_2-1\right)+\Lambda +\left({M}_n-1\right)+{N}_1\times {N}_2\times \Lambda \times {N}_i$$

*M*_*i*_: risk factor scores for *S* ≥ 1; *N*_*i*_: risk factor scores for *S* < 1

(IV) Individual risk prediction score of EOCRC (*I*)

$$I={Q}_{EOCRC}\times \theta$$

*Q*_*EOCRC*_: The incidence of EOCRC.

### Statistical analysis

We performed sensitivity analysis on variables that were significant. Only variables that showed significance (*P* < 0.05) in the fixed-effects model combined with the random-effects model were considered stable. These eligible variables were then included in the risk scoring system. Variables that exhibit significance in only one model will be excluded from the risk system since they were considered to be unstable [19]. The risk of publication bias was calculated using Egger’s test. Simulated data of 10,000 subjects were randomly generated using the binomial distribution function method. The individual risk prediction scores of EOCRC (*I*) were calculated after substitution of the simulated data into the Rothman–Keller model. Statistical analysis was performed using STATA 15.1 software (Stata Corporation, College Station, TX, USA) and RStudio software (version 1.4).

## Results

### Literature selection

**Fig. 1:**
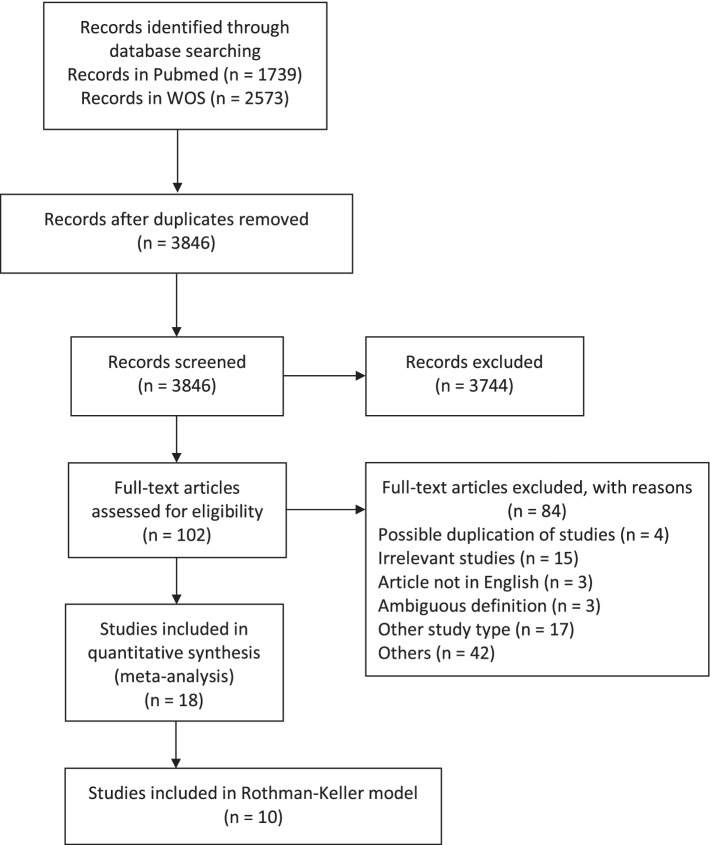
The flow chart of the study selection and identification

The literature search identified 4312 publications, of which 3846 were unique studies. A total of 3744 publications were excluded because they did not meet the inclusion criteria. After screening the full text of the remaining 102 studies, 18 articles were included in the meta-analysis, four of which were new compared to the previously published meta-analysis. Ten studies were used for the construction of the risk appraisal model as they provided baseline data of case and control groups, containing a total of 32,843 cases and 25,806,408 controls. Figure 1 shows the flow chart of the study selection and identification.

### Risk factors for EOCRC

As shown in Table 1, based on the combined ORs and *P*-values, we identified nine core risk factors influencing the development of EOCRC, namely male sex, Caucasian ethnicity, family history of CRC, sedentary behavior, alcohol intake, obesity, diabetes, IBD, and high intake of red meat. However, the use of NSAIDs or aspirin and high intake of dessert were excluded due to the lack of sufficient studies (*n* = 2). The results of the fixed-effects and random-effects models showed that the joint effect of smoking, hypertension, hyperlipidemia, and educational level was unstable (*P* for fixed-effects model < 0.05 while *P* for random-effects model > 0.05). Thus, these factors were excluded in the Rothman–Keller model. In addition, although high intake of processed meat did not show a significant association in the meta-analysis, it was included in the construction of the prediction model as a potential factor for EOCRC because it showed a significant trend (OR = 1.24, 95% CI = 0.99–1.55). No publication bias was found using Egger’s test (*P*>0.05).

**Table 1:** Results of the combined assessment of various risk factors

Risk factors	No.^a^	Cases, n Exposure (+/−)	Controls, n Exposure (+/−)	Reference for data mining	Fixed effect model		Random effects model			
					OR	95%CI	*P*	OR	95%CI	*P*
Male sex	9	8533/8073	5,013,326/6,907,255	[7, 9, 22–27]						
Yes					1.16	1.12–1.20	0.001	1.18	1.02–1.37	0.031
No					1			1		
Caucasian ethnicity	6	16,790/7037	15,145,866/10,614,153	[8, 22–25, 27]						
Yes					1.77	1.72–1.83	0.001	1.41	1.16–1.73	0.001
No					1			1		
Family history of CRC	9	2350/8496	308,951/11,502,258	[7, 9, 22, 23, 25, 27]						
Yes					7.03	6.67–7.41	0.001	4.13	1.68–10.13	0.002
No					1			1		
Sedentary	5	294/1167	592/3175	[7, 9, 23, 28]						
Yes					1.26	1.06–1.48	0.007	1.33	1.05–1.70	0.02
No					1			1		
Smoking	9	10,860/16,576	8,367,277/17,375,989	[7, 8, 22–25, 27, 28]						
Yes					1.44	1.40–1.47	0.001	1.23	0.93–1.64	0.039
No					1			1		
Alcohol^b^	5	2852/5403	2,833,801/8,971,210	[7, 9, 23, 27, 28]						
Yes					1.66	1.58–1.75	0.001	1.52	1.28–1.80	0.001
No					1			1		
Obesity	10	12,195/15,887	8,114,710/17,676,715	[8, 22–24, 26, 27]						
Yes					2.30	2.25–2.36	0.001	1.42	1.13–1.78	0.003
No					1			1		
Hypertension	5	4794/22,980	2,886,828/22,846,354	[8, 22, 25–27]						
Yes					1.49	1.44–1.54	0.001	1.36	0.84–2.21	0.213
No					1			1		
Hyperlipidemia	5	5602/22,172	2,125,036/23,608,146	[8, 22, 25–27]						
Yes					2.11	2.05–2.19	0.001	1.39	0.94–2.06	0.099
No					1			1		
Diabetes	8	1663/25,143	494,375/13,511,324	[7–9, 22–26]						
Yes					1.47	1.39–1.55	0.001	1.25	1.04–1.50	0.019
No					1			1		
IBD	3	936/9716	396,324/11,446,050	[25–27]						
Yes					3.46	3.23–3.71	0.001	3.20	1.60–6.41	0.001
No					1			1		
Red meat	3	1214/1081	1260/1945	[7, 9, 23]						
Yes					1.12	1.07–1.17	0.001	1.12	1.07–1.17	0.001
No					1			1		
Processed meat	4	658/573	872/1443	[7, 9, 23, 28]						
Yes					1.10	1.03–1.19	0.007	1.24	0.99–1.55	0.064
No					1			1		
High school education	4	1572/2705	2174/4441	[7, 9, 23, 25]						
Less					1.14	1.09–1.20	0.001	1.49	0.78–2.87	0.230
More					1			1		

^a^Number of studies for meta-analysis

^b^Pooled effect estimates to obtain the highest exposure defined category compared with the lowest

### Parameters of the risk appraisal model

The proportion of exposed individuals in the control group was used as an estimate of the overall population exposure rate (*P*_*i*_). The RR values (*RRs*) in the Rothman–Keller model were replaced by the combined OR values (*OR*_*i*_) from the meta-analysis. The parameters of the EOCRC risk appraisal model are shown in Table 2.

**Table 2:** Risk assessment model parameters of EOCRC

Risk factor	*RR*_*i*_	*P*_*i*_	*PAR%*	*ρ*	*S*
Male					
Yes	1.18	0.4206	0.0704	0.9296	1.0970
No	1	0.5794			0.9296
Caucasian ethnicity					
Yes	1.41	0.5880	0.1942	0.8058	1.1361
No	1	0.4120			0.8058
Family history of CRC					
Yes	4.13	0.0262	0.0757	0.9243	3.8175
No	1	0.9738			0.9243
Sedentary					
Yes	1.33	0.1572	0.0493	0.9507	1.2644
No	1	0.8428			0.9507
Alcohol					
Yes	1.52	0.2401	0.1110	0.8890	1.3513
No	1	0.7599			0.8890
Obesity					
Yes	1.42	0.3146	0.1167	0.8833	1.2543
No	1	0.6854			0.8833
Diabetes					
Yes	1.25	0.0353	0.0087	0.9913	1.2391
No	1	0.9647			0.9913
IBD					
Yes	3.2	0.0335	0.0686	0.9314	2.9806
No	1	0.9665			0.9314
Red meat					
Yes	1.12	0.3931	0.0451	0.9549	1.0695
No	1	0.6069			0.9549
Processed meat					
Yes	1.24	0.3767	0.0829	0.9171	1.1372
No	1	0.6233			0.9171

### Calculation of the EOCRC individualized risk assessment

Individualized combined risk scores (*I*) were calculated based on the parameters in Table 2 (Formula III and IV). For example, a male subject (subject A) younger than age 50 years (S = 1.0970), Caucasian (S = 1.1361), with a family history of CRC (S = 3.8175), history of alcohol consumption (S = 1.3513), diabetes (S = 1.2391), and high intake of red meat (S = 1.0685), but without the characteristics of IBD (S = 0.9314), sedentary lifestyle (S = 0.9507), obesity (S = 0.8833), and high intake of processed meat (S = 0.9171). Accordingly, the combined risk score (*θ*) of subject A = (1.0970–1) + (1.1361–1) + (3.8175–1) + (1.3513–1) + (1.2391–1) + (1.0685–1) + 0.9314 × 0.9507 × 0.8833 × 0.9171 = 4.427. A study based on the U.S. Surveillance, Epidemiology, and End Results (SEER) database reported a 0.12% prevalence of EOCRC [8]. Therefore, the individual risk prediction score of EOCRC (*I*) for subject A = 0.12% * 4.427 = 0.531%.

### Level of EOCRC risk assessment

**Fig. 2:**
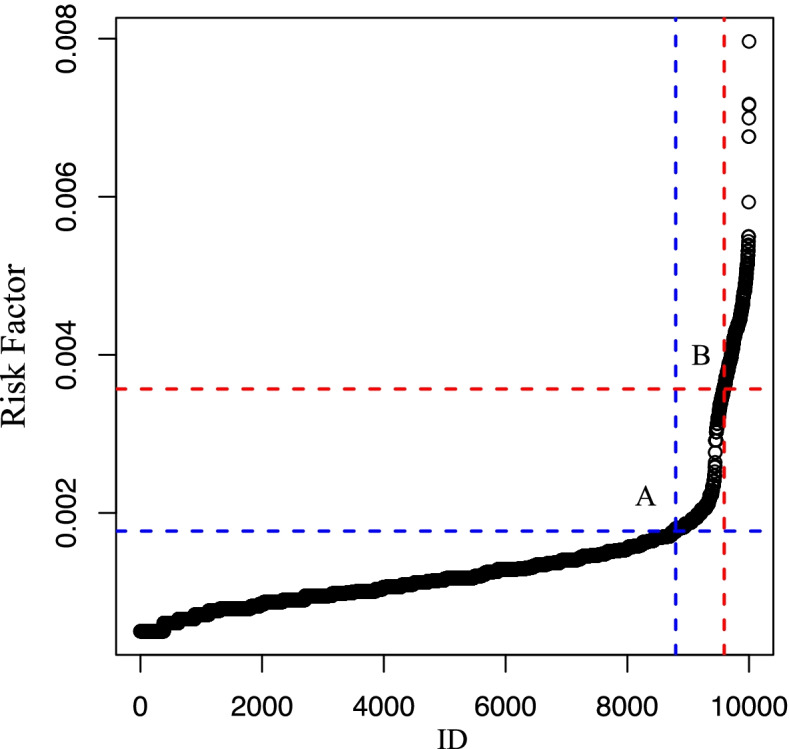
The predictive analysis of Rothman–Keller model for EOCRC

Figure 2 and Supplementary Table 1 show the individual risk scores of 10,000 simulated subjects sorted in ascending order. The 8795th (*I* = 0.0018, point A) and 9591st (*I* = 0.0036, point B) positions were selected as the nodes for the level of EOCRC risk assessment. Individual risk prediction scores (*I*) of 0 to 0.0018, 0.0018 to 0.0036, and 0.0036 or higher were considered low, medium, or high risk. Accordingly, Subject A was in a high-risk group, and we strongly recommend that he should receive health education and clinical screening.

## Discussion

Although CRC is still relatively rare in the younger aged population (0.12%), the alarming increasing in EOCRC patients cannot be ignored [29, 30]. The clinical cases, molecular, and familial features of EOCRC strongly suggest that it may be a separate disease rather than a subset of CRC [31, 32]. It is estimated that there may be a 30–120% increase in young colorectal cancer patients by 2030 based on current trends [33]. In addition, most EOCRCs are insidious and have a worse prognosis compared to late-onset CRC, which undoubtedly increases the difficulty of diagnosis and disease prevention [34]. Although annual screening is strongly recommended for individuals with a family history of CRC in first-degree relatives, the lack of subjective knowledge about the high risks of CRC and the negative attitudes towards clinical screening are the main reasons why most young adults are reluctant to undergo screening, which undoubtedly increases the difficulty of primary prevention in high-risk groups [35, 36]. It is important to construct a risk assessment system based on clinical or behavioral factors. Previous studies have developed clinical prediction models based on colonoscopy or stool test results [37–39]. Although such tests identify a subset of patients that may benefit from them, most predictive tools require specialized assessment by clinicians and are not suitable for prospective population screening [40]. In addition, precise cancer screening or modified screening regimens based on risk-stratification may allow adults to benefit more from CRC screening than conventional age-based strategies [41]. Therefore, we prefer to build a risk prediction system in which subjects can independently participate, and contributes to encouraging young adults to screen for potential disease probability before visiting the clinic.

Compared to the previous meta-analysis [14], we identified significant associations between sedentary, IBD, diabetes, high intake of red meat and processed meat, and the development of EOCRC, as we included more original studies. Although the correlation between the high intake of processed meats and EOCRC was not statistically significant, the trend it exhibited was equally alarming [42]. The role of non-genetic factors, especially dietary factors, in the pathogenesis of EOCRC should not be ignored. Several studies have also reported a positive association between reduced intake of folate, fiber, citrus fruits, and greater risk of EOCRC [7, 9]. Unfortunately, most studies do not include regional factors as one of the variables, which prevents us from understanding the contribution of urban-rural or regional differences to the incidence of EOCRC, although this appears to be potentially relevant at present [43–45]. Our study constructed a more accurate model based on a meta-analysis that considered and quantified interactions among risk factors, providing a prediction system for individuals under the age of 50 years. In contrast to non-modifiable factors such as sex and race, most risk factors we identified were common and changeable behavioral factors, such as sedentary lifestyle, high intake of red meat and processed meat, and alcohol consumption. This means that young subjects who are alerted may reduce the incidence of EOCRC by modifying their diet or daily behavior patterns. As far as we know, most people appear to be more receptive to modifying their personal risk through diet and exercise [16]. Despite the prevalence of these factors among the general CRC population, we can still find some evidence on how these variables influence the development of EOCRC. It is well known that family history of cancer, obesity, sedentary lifestyle and high consumption of high calorie, high fat, high sucrose diet are the key factors in CRC [46]. The prevalence of obesity has increased in the USA, especially among young patients, which may play a role in reducing the age of CRC onset [33]. Similar problems exist in other countries [47, 48]. The prevalence of known risk factors such as diabetes, smoking, and alcohol consumption continues to rise [49–51], and these high-risk behaviors in young adults increase the incidence of EOCRC despite measures already in place to counter. Patients with longstanding IBD have a two to three times increased risk of CRC, especially when diagnosed at an early age [52]. Approximately 2–5% of the general CRC population is affected by hereditary cancer syndromes. However, this appears to be higher (22%) in patients diagnosed with EOCRC [2]. Besides, the increasing global prevalence of non-Mediterranean Western dietary patterns, characterized by a high intake of red and processed meats, among the young population has undoubtedly increased the burden of EOCRC [53]. Therefore, there is a significant need to enhance health education to control these potential risk factors.

This study had some limitations. First, although we generated a group of random data sets using a binomial distribution method, there is a lack of evidence supporting and validating results from multicenter, large-scale, and real-world studies. Second, studies on risk factors for EOCRC are still very limited and lead to the exclusion of other potential risk factors from the model construction due to insufficient statistical power.

## Conclusions

We established a risk appraisal model for EOCRC based on meta-analysis and the Rothman–Keller model to provide personalized health education and screening for individuals with different risk levels, which can be used for primary prevention of CRC and to help reduce the incidence of EOCRC.

## Supplementary Information

**Additional file 1.** The individual risk scores of 10,000 simulated subjects.

## Data Availability

All data generated or analysed during this study are included in this published article [and its supplementary information files].

## Abbreviations

EOCRC: Early-onset colorectal cancer
CRC: Colorectal cancer
WOS: Web of Science
SEER: Surveillance, Epidemiology, and End Results database
